# Arabinoxylans, inulin and *Lactobacillus reuteri* 1063 repress the adherent-invasive *Escherichia coli* from mucus in a mucosa-comprising gut model

**DOI:** 10.1038/npjbiofilms.2016.16

**Published:** 2016-07-27

**Authors:** Pieter Van den Abbeele, Massimo Marzorati, Melanie Derde, Rosemarie De Weirdt, Vermeiren Joan, Sam Possemiers, Tom Van de Wiele

**Affiliations:** 1 Center of Microbial Ecology and Technology (CMET), Ghent University, Ghent, Belgium; 2 ProDigest, Ghent, Belgium

## Abstract

The microbiota that colonises the intestinal mucus may particularly affect human health given its proximity to the epithelium. For instance, the presence of the adherent-invasive *Escherichia coli* (AIEC) in this mucosal microbiota has been correlated with Crohn’s disease. Using short-term screening assays and a novel long-term dynamic gut model, which comprises a simulated mucosal environment (M-SHIME), we investigated how (potential) pro- and prebiotics may repress colonisation of AIEC from mucus. Despite that during the short-term screening assays, some of the investigated *Lactobacillus* strains adhered strongly to mucins, none of them competed with AIEC for mucin-adhesion. In contrast, AIEC survival and growth during co-culture batch incubations was decreased by *Lactobacillus rhamnosus* GG and *L. reuteri* 1063, which correlated with (undissociated) lactic acid and reuterin levels. Regarding the prebiotics, long-chain arabinoxylans (LC-AX) lowered the initial mucin-adhesion of AIEC, while both inulin (IN) and galacto-oligosaccharides (GOS) limited AIEC survival and growth during batch incubations. *L. reuteri* 1063, LC-AX and IN were thus retained for a long-term study with the M-SHIME. All treatments repressed AIEC from mucus without affecting AIEC numbers in the luminal content. As a possible explanation, *L. reuteri* 1063 treatment increased lactobacilli levels in mucus, while LC-AX and IN additionally increased mucosal bifidobacteria levels, thus leading to antimicrobial effects against AIEC in mucus. Overall, this study shows that pro- and prebiotics can beneficially modulate the *in vitro* mucosal microbiota, thus limiting occurrence of opportunistic pathogens among those mucosal microbes which may directly interact with the host given their proximity to the epithelium.

## Introduction

Along the intestinal tract, the host epithelium is covered by a protective mucus layer that contains specific microbes^1^ and several factors contribute to a distinct microbiota in the luminal content versus the mucus layer.^2^ First, the host selects microbes that colonise mucus by producing defence molecules such as antimicrobial peptides (AMPs) and IgA.^3^ Selection also occurs through oxygen diffusion from the blood towards the gut, resulting in an oxygen gradient along the mucus. The unique mucosal microbiota composition is further determined by bacterial factors such as mucus adhesion^4^ and mucin degradation.^5^ The resulting mucosal microbiota as such possesses a colonisation resistance against opportunistic pathogens. This includes local excretion of antimicrobial compounds,^6^ stimulation of the host immune system,^7^ production of metabolic compounds that lower the pH^8^ and competition with pathogens for nutrients^9^ and adhesion sites.^10^

A disruption of the mucosal microbial community may have adverse implications for human health. Besides an altered interaction with the immune system, a disrupted mucosal microbiota may result in increased colonisation by (potential) pathogens correlated with infectious or chronic gastrointestinal diseases. As an example, the mucosal microbiota of Crohn’s disease (CD) patients is characterised by the dominance of potentially harmful microbes, in particular *Escherichia coli*, over beneficial microbes such as *Faecalibacterium prausnitzii* and *Roseburia* sp.^11,12^ In fact, the adherent-invasive *Escherichia coli* (AIEC), a strain proposed as a causative agent of CD, can strongly adhere to and invade intestinal epithelial cells in patients by means of a protease called Vat-AIEC.^13,14^

While *in vivo* studies are restricted to end-point measurements regarding mucosal microbes,^1^ *in vitro* assays allow to study mechanisms of microbial adhesion. These assays include adhesion experiments to mucus,^15^ mucins,^16^ colonic tissue^17^ and cell lines.^18^ Because these models only provide short-term information and ignore the interaction between luminal and mucosal microbes, we modified a dynamic *in vitro* model for the luminal microbiota by incorporating mucin-covered microcosms (M-SHIME). The validation of this M-SHIME model followed from the more representative colonisation of *Lactobacillus* sp.^19^ Furthermore, high-resolution phylogenetic characterisation showed that the simulated mucosal microbiota was, in correspondence with *in vivo* studies, enriched with Firmicutes sp. belonging to *Clostridium* clusters IV and XIVa.^20^ The same study showed that the *in vitro* mucosal environment is necessary to avoid wash-out of relevant surface-attached microbes.

As reinforcing the mucosal microbiota may be a strategy to restore the host-microbe interaction when disturbed, the objective of this study was to evaluate the efficacy of several strategies to enhance the mucosal microbiota in repressing opportunistic pathogens from the mucosal environment. To demonstrate the proof-of-principle, we used AIEC as a model opportunistic pathogen, as AIEC is known to strongly adhere to mucus in CD patients, while the ampicillin/erythromycin-resistant reference strain LF82^21^ enables straightforward detection of viable AIEC cells in the background of a mixed intestinal microbiota. Using short-term screening assays, we evaluated how selected pre- and probiotics may inhibit the initial adhesion of AIEC to intestinal mucins, as well as the survival/growth of AIEC. A selection of treatments was then applied in the M-SHIME to investigate whether AIEC was repressed in a simulated mucosal environment during a long-term interaction with the resident luminal and mucosal microbiota of a single donor.

## Results

### Short-term mucin-adhesion experiments

**Figure 1:**
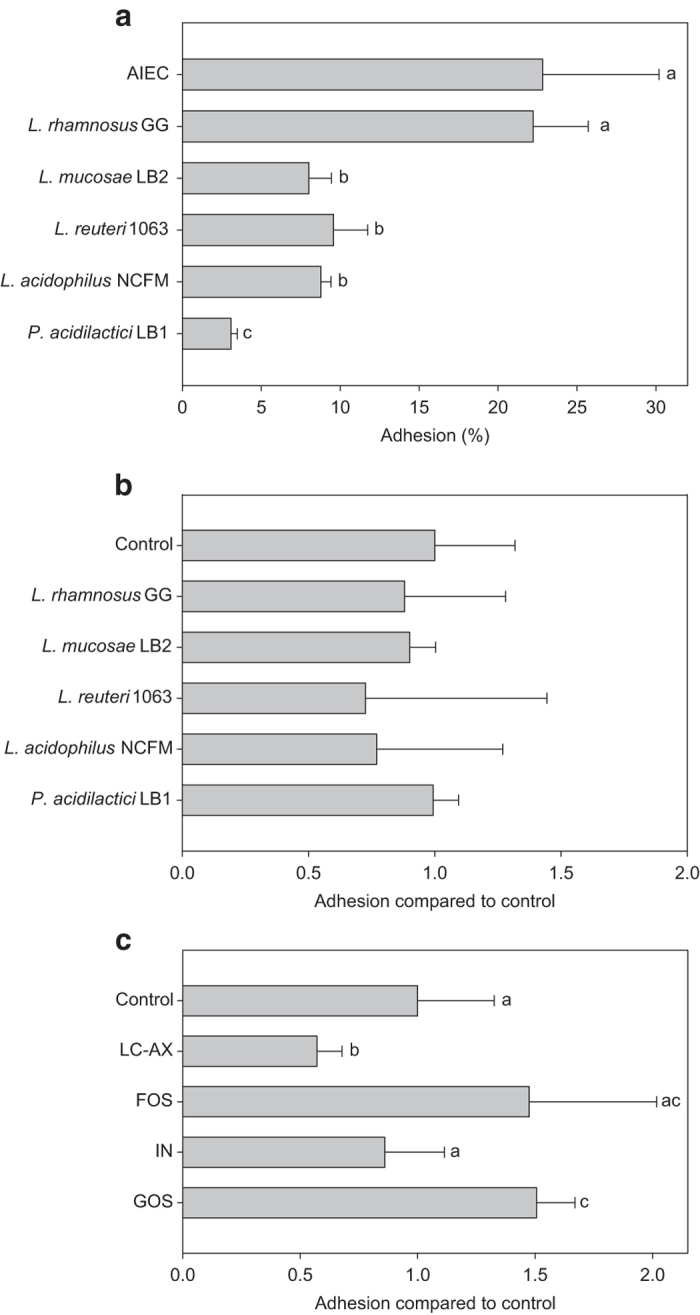
(**a**) Mean intrinsic mucin-adhesion capacity of AIEC (±s.d.) (*n*=16) and several *Lactobacillus* strains (*n*=4), expressed as a ratio of the amounts of adhered bacteria compared with initially added bacteria (10^8^ cells per ml). (**b**) Adhesion of AIEC in the presence of several *Lactobacillus* strains, as a ratio compared with the control where only AIEC was added (*n*=4). (**c**) Adhesion of AIEC in the presence of several prebiotic compounds (LC-AX; *n*=7, FOS; *n*=4, IN; *n*=4 and GOS; *n*=4), as a ratio compared with the control where only AIEC was added (*n*=11). Values indicated with a different superscript are significantly different (*P*⩽0.05; a, b or c).

As inhibition of AIEC adhesion by *Lactobacillus* strains may depend on competition for mucin-binding sites, the intrinsic adhesion capacity of the probiotic strains was evaluated in a first experiment. This showed that the strains significantly differed in their intrinsic adhesion capacity (Figure 1a). While the adhesion capacity of *Lactobacillus rhamnosus* GG was strong and comparable to that of AIEC (~20%), *Pediococcus acidilactici* LB1 adhered poorly (~3%). Adhesion of *L. mucosae* LB2, *L. reuteri* 1063 and *L. acidophilus* NCFM was intermediate (~10%). However, none of the tested *Lactobacillus* strains lowered adhesion of AIEC in competition experiments, as compared with the AIEC control without probiotics (Figure 1b).

In contrast, two prebiotic compounds interfered with the initial adhesion of AIEC to mucins (Figure 1c). While fructo-oligosaccharides (FOS) and inulin (IN) had no influence, long-chain arabinoxylan (LC-AX) significantly inhibited adhesion of AIEC (*P*=0.008). Finally, in presence of galacto-oligosaccharides (GOS), the adhesion of AIEC was significantly higher (*P*=0.009).

### Short-term survival/growth inhibition assay

On inoculation of AIEC in the nutritional medium, a good growth was monitored with a factor 60 increase in the control after 24 h. All strains tested, except *L. mucosae* LB2, inhibited AIEC survival/growth (Table 1). AIEC numbers after 24-h incubation were significantly lower—as compared with the control—in the presence of *P. acidilactici* LB1, *L. acidophilus* NCFM*, L. rhamnosus* GG and *L. reuteri* 1063. For these last two strains, a complete eradication of AIEC below detection limit was observed. As AIEC is able to produce both acetate and ammonium, these metabolites are potential markers for AIEC growth, even if it has to be taken into account that these metabolites can also remain high due to growth of other species. Acetate levels correlated with AIEC growth/survival, with the exception of the co-culture with *L. reuteri* 1063, in which acetate levels remained high despite the fact that AIEC was eradicated. Ammonium levels only decreased for the treatments that eradicated AIEC below detection limit, i.e., *L. reuteri* 1063 and *L. rhamnosus* GG. To provide an explanation for the observed antimicrobial effects, the pH decrease and concentrations of D/L-lactic acid (and its antimicrobial fraction, i.e., undissociated lactic acid), 3-HPA and 1,3-PDO (markers for reuterin) were determined. It followed that a decrease in AIEC numbers corresponded with lower pH and increased levels of undissociated lactic acid. The strong antimicrobial effect of *L. reuteri* 1063 was plausibly due to the combined action of undissociated L-lactic acid and reuterin (approximate increase of 3-HPA and 1,3-PDO), while in the case of *L. rhamnosus* GG it was related to high levels of undissociated L-lactic acid.

The indirect antimicrobial effect of oligo- and polysaccharides has been evaluated in relation to the production of metabolites resulting from microbial catabolism. In this respect, the nutritional medium was inoculated with AIEC and also with 5×10^6^ c.f.u. per ml of an ascending colon suspension from a SHIME system (control B). It was found that this mixed microbiota already exerted a vast inhibitory effect on AIEC growth (approximately only factor 0.27 increase after 24 h in control B in contrast to control A without mixed microbiota, which showed a 57.2±19.7-fold increase) (Table 2). Both GOS and IN further enhanced the inhibitory effect of this mixed microbiota against AIEC, while LC-AX did not affect AIEC. For GOS, the decrease in AIEC levels coincided with a lower pH and more undissociated lactic acid, while for IN higher levels of 1,3-PDO and thus potentially more reuterin were observed as opposed to the other prebiotics. Despite elevated levels of undissociated lactic acid, FOS inhibited the growth of AIEC less than what is seen for control B. Given the metabolic activity of the mixed microbiota, acetate and ammonium were no optimal markers for AIEC growth. Together with propionate, butyrate and the total SCFA levels, they rather indicated the growth of the mixed intestinal microbiota.

### Long-term M-SHIME study

Based on initial screenings, three treatments were applied in a follow-up M-SHIME experiment (i.e., *L. reuteri* 1063, LC-AX and IN) with the aim of understanding whether AIEC is repressed from a simulated mucosal environment during a long-term interaction with the resident luminal and mucosal microbiota.

**Figure 2:**
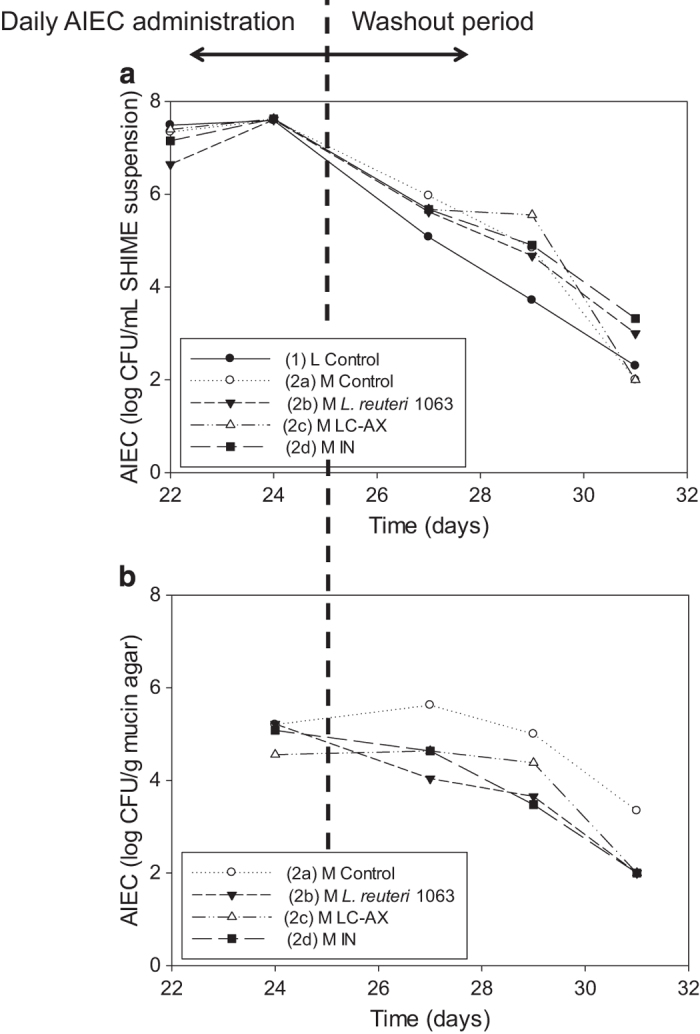
The AIEC numbers in the luminal (log c.f.u. per ml suspension) (**a**) and mucosal environment (log c.f.u. per g mucin agar) (**b**) of five ascending colon compartments. The first unit consisted of the conventional setup that only contains luminal microbes (L-SHIME) (1), whereas the other four units were modified by incorporating a mucosal compartment (=M-SHIME). The L-SHIME and the first M-SHIME were fed the normal nutritional SHIME medium (2a), while the other three were treated with *L. reuteri* 1063 (2b), LC-AX (2c) and IN (2d), respectively. AIEC was inoculated on day 22, 23, 24 and 25 at ~2.10^8^ c.f.u. per ml. Samples were collected before renewal of mucus on day 24, 27, 29 and 31.

The AIEC numbers (c.f.u. per ml) in the luminal environment were similar between the different ascending colon units of the M-SHIME, but tended to be lower in that of the L-SHIME, which lacks a mucosal environment (Figure 2a). In contrast, AIEC counts within the mucosal environment were at least 1-log unit lower as compared with the control as a result of the treatments in the M-SHIME (Figure 2b). On inoculation of AIEC (on day 22, 23, 24 and 25), LC-AX immediately decreased AIEC in the mucus (day 24). During further treatment, supplementation of *L. reuteri* 1063, LC-AX and IN was similarly effective in lowering AIEC numbers in mucus. After the AIEC administration period, the average decrease of AIEC in the treated mucus (average calculated on the concentrations measured on day 27, 29 and 31) was 1.43±0.14 log c.f.u. per g mucus for *L. reuteri* 1063, 0.98±0.36 log c.f.u. per g mucus for LC-AX and 1.29±0.27 log c.f.u. per g mucus for IN.

As assessed by DGGE, the overall bacterial community composition rather clustered according to the location (mucus or lumen) than according to treatment, caused by different abundances of subdominant microbes (Supplementary Figure 1A), suggesting only a minor role of abundant gut anaerobes in the inhibitory effect towards AIEC. As prebiotic compounds are known to target subdominant bacteria belonging to lactobacilli and especially bifidobacteria,^22^ the quantities of these groups were determined in the luminal and mucosal environment (Table 3). Supplementation of *L. reuteri* 1063, LC-AX and IN increased lactobacilli levels in both lumen and mucus. LC-AX and IN also increased the levels of *Bifidobacterium* spp. in mucus. Furthermore, LC-AX and IN altered the *Bifidobacterium* composition. While the control and *L. reuteri* 1063-treated vessels were dominated by *B. bifidum,* LC-AX-stimulated *B. longum* in lumen and mucus, and *B. adolescentis* in mucus. IN led also to a delayed stimulation (i.e., day 29) of *B. adolescentis* in mucus and lumen (Supplementary Figure 1B). In addition, *L. reuteri* 1063 elevated lactic acid and reuterin (~1,3-PDO) levels, while LC-AX and IN only increased reuterin production (~1,3-PDO) (Table 3).

## Discussion

In this study, we provide an *in vitro* proof-of-principle that pre- and probiotic strategies can beneficially modulate the mucosal microbiota and decrease the mucosal colonisation of an opportunistic pathogen (i.e., *Escherichia coli* AIEC). Because the mucosal microbes may directly interact with the host, lowering the abundance of opportunistic pathogens in the mucosal compartment may be more relevant than lowering their abundance in the intestinal content. We used the AIEC as a model opportunistic pathogen because it is known to adhere strongly to mucus and because its reference strain LF82 can be easily detected in a mixed microbiota.^21^ As the ileal microbiota is difficult to simulate *in vitro* due to the lack of a representative inoculum and the difficulty of simulating the host factors that govern ileal composition (e.g., secretion of AMPs, high flow through), we simulated the ascending colon considering that AIEC are associated both with ileal and colonic disease phenotypes.^14^ After short-term screening assays, a selection of pro- and prebiotics was applied in a dynamic *in vitro* model (M-SHIME) that allows to study both the luminal and mucosal intestinal microbiota.^19^ This simulated mucosal environment avoids wash-out of specific surface-associated microbes.^20^ As AIEC also benefits from mucus adhesion to colonise the human intestine, this novel model allowed studying the colonisation of AIEC in a more representative manner compared with earlier models.

The initial mucin-adhesion capacity of AIEC was strong and it was comparable to that of well-known mucus colonisers such as *L. rhamnosus* GG^23^ and *L. reuteri* 1063^4^ (Figure 1a). The mucin-adhesion of AIEC was confirmed during the long-term experiment, where AIEC was an abundant member of the mucosal microbiota, even 6 days after its last inoculation (day 31; Figure 2b). The ability of AIEC to colonise the intestinal surface has been attributed to active motility through flagella which also regulate expression of type 1 pili.^24^ For AIEC, mucus colonisation is crucial to adhere to and invade host cells.^13,25^

*L. reuteri* 1063, LC-AX and IN specifically lowered AIEC numbers in the simulated mucosal environment of the M-SHIME, while they did not affect AIEC numbers in the luminal content. As a possible explanation, supplementation of IN and LC-AX (during 8 days prior to AIEC inoculation) altered the resident mucosal microbiota, especially subdominant groups (lactobacilli and bifidobacteria). Not only did LC-AX and IN increase the mucosal counts of lactobacilli and bifidobacteria, they also altered the species composition of the bifidobacteria. As shown by DGGE, initially the dominant mucosal *Bifidobacterium* species was *B. bifidum*, IN specifically stimulated *B. adolescentis* in mucus and LC-AX specifically stimulated both *B. longum* and *B. adolescentis* in mucus. *B. longum* has been shown to possess a stronger antimicrobial activity against *Escherichia coli* compared with *B. bifidum.*^26^ Also *B. adolescentis* was shown to be very effective in combating *Escherichia coli* compared with *B. bifidum*^27^ but also compared with many other *Bifidobacterium* and *Lactobacillus* species.^28^ While antimicrobial factors are possibly too diluted in the intestinal content to be effective, the mucosal environment may allow trapping of antimicrobial factors, thereby repressing AIEC. Especially the spatial heterogeneity introduced by the biofilm on top of the mucin layer may result in local accumulation of e.g., acids produced by lactobacilli or bifidobacteria.

Although the short-term screening assays are inevitably confounded by drawbacks such as pH drops, nutrient limitation and accumulation of metabolites, they allowed to demonstrate that both the conversion of glycerol to reuterin and the undissociated lactic acid production are processes that exert distinct antimicrobial effects on AIEC (Tables 1 and 2). As shown for LGG and GOS, the antimicrobial effect of lactic acid is dependent on a concomitant pH decrease as this enhances the portion of undissociated lactic acid. Only undissociated lactic acid can pass through the cell wall after which it exerts an antimicrobial effect by releasing protons in the cytoplasm of AIEC. While the pH was controlled in the intestinal lumen of the M-SHIME (pH=5.6–5.9), the mucosal pH may have decreased below 5.6 resulting in higher mucosal levels of undissociated lactic acid (>1.79%). Further, for *L. reuteri* 1063 and the mixed intestinal microbiota, the presence of the glycerol conversion products, 3-HPA and 1,3-PDO, indicated the production of the potent, broad-spectrum antimicrobial reuterin capable of inhibiting growth of many other microbial species, including *Escherichia* spp.^29–32^ As glycerol levels are higher in CD patients^33^ and in the upper digestive tract, the conversion of glycerol to reuterin may be particularly relevant for AIEC eradication.

Another mechanism that may result in lower mucosal AIEC counts is an adhesion inhibition of AIEC to intestinal mucins. However, among all (potential) pro- and prebiotics tested (even the strongly adherent probiotics), LC-AX was the only treatment that lowered the initial AIEC adhesion (Figure 1b,c). Also during the long-term M-SHIME study, only LC-AX limited the initial colonisation of the mucosal environment by AIEC (Figure 2b). These results are concordant with earlier studies showing that this specific LC-AX can lower the initial mucin-adhesion of a wide variety of bacterial groups including coliforms.^16^ The latter study demonstrated that the underlying reason may be that LC-AX increase the viscosity. Further, physical adhesion inhibition may occur by binding of microbes to fibres instead of surface receptors.^34^ LC-AX-type polysaccharides may thus be interesting compounds to modulate the initial bacterial adhesion to mucins.

Despite the fact that the mucosal environment was partly (50%) renewed every 2 days, specific species cross-contaminated between old and new mucin-microcosms, resulting in a distinct microbiota in the luminal and mucosal environment (Supplementary Figure 1). In previous studies with the M-SHIME, where the mucosal environment was not renewed, even stronger differences between the mucosal and luminal microbiota were observed on day 1 (~60% similarity)^19^ or day 3 after start-up (only ~15% similarity).^20^

In conclusion, we showed that the M-SHIME technology—using mucin-covered microcosms—provided a detailed insight in the long-term *in vitro* microbial colonisation of AIEC, an opportunistic pathogen and abundant mucosal microbe. Moreover, it allowed to evaluate the colonisation of a simulated mucus layer in the presence of a resident mucosal and luminal intestinal microbiota. It has to be considered that in this type of studies, the relevance of the data in terms of potential interindividual variability (i.e., different effect of the test products due to a different composition of the gut microbiota) may be questionable. Our aim was to present a technology platform that might be applied on other disease-causing microbes such as food-borne pathogens and to provide evidences on the potential of several pre- and probiotic strategies to repress AIEC from the mucosal compartment. Here we showed—with the microbiota from one donor—that such repression may occur via different mechanisms including the production of reuterin, undissociated lactic acid or adhesion inhibition. The evaluation of the role of the microbiota from different donors on these mechanisms can be an interesting future line of research. We also showed that the incorporation of a mucosal environment in dynamic gut models may be a powerful tool to obtain a more realistic view on processes that drive the gastrointestinal microbiome.

## Materials and methods

### Preparation of growth media and bacterial suspensions

Unless stated otherwise, chemicals were obtained from Sigma (Bornem, Belgium). Cosucra (Warcoing, Belgium) provided FOS with a purity of 96% and a degree of polymerisation (DP) between 2 and 20 (Fibrulose F97) and IN with a purity of 92% and a DP between 3 and 60 (Fibruline instant). Friesland Campina Domo (Amersfoort, the Netherlands) provided GOS with a purity of 98% and a DP between 3 and 8 (Vivinal GOS, Friesland Campina Domo). BioActor (Maastricht, the Netherlands) provided water-extractable LC-AX with a purity of 60%, a degree of substitution of 0.7 and an average DP⩾60.

Ampicillin/erythromycin-resistant AIEC LF82, isolated from a chronic ileal lesion of a CD patient, was used as the AIEC reference strain.^21^ Following strains were used during this study: *L. rhamnosus* GG (LMG 18243), *L. reuteri* 1063 (ATCC 53608), *L. acidophilus* NCFM (Danisco, Brugge, Belgium), *L. mucosae* LB2^19^ and *P. acidilactici* LB1.^19^ Pure cultures of *Lactobacillus* sp. were grown overnight in MRS medium (Oxoid, Cambridge, UK), while AIEC was grown in BHI medium (Oxoid), both at 37 °C under aerobic conditions.

The nutritional medium of the M-SHIME consisted of (in g/l) arabinogalactan (1.0), pectin (2.0), xylan (1.0), starch (3.0), glucose (0.4), yeast extract (3.0), peptone (1.0), mucin (4.0) and cystein (0.5). Glycerol (1.0) was added to enable *L. reuteri* among others to produce reuterin, a bacteriocin.^35^ Pancreatic juice contained (in g/l) NaHCO_3_ (12.5), bile salts (6.0) (Difco, Bierbeek, Belgium) and pancreatin (0.9).

### Short-term AIEC mucin-adhesion assay

In a first experiment, the intrinsic adhesion capacity of AIEC and the *Lactobacillus* strains to mucins was determined. Moreover, inhibition of AIEC adhesion by these (potential) probiotics and prebiotics (FOS, IN, GOS and LC-AX) was evaluated. The mucin-adhesion assay was performed as described previously.^16^ Briefly, an overnight culture was diluted in fresh growth medium (1:10) and allowed to grow for another 3 h. Bacterial cells were washed three times with filter-sterilised 0.1 M phosphate-buffered saline (PBS) at pH 5.9 and diluted to a final density of ~10^8^ cells per ml (using flow cytometry on live–dead staining). Immediately thereafter, 1 ml of bacterial suspension and 1 ml of PBS were added to 12-well plates covered with 1.2 ml mucin agar. Mucin agar was prepared by boiling distilled H_2_O containing 5% gastric porcine mucin type II (Sigma) and 1% agar. The pH was adjusted to 6.8 with 10 M NaOH (~350 μl per 100 ml). Bacteria were allowed to adhere to this mucin layer under anaerobic conditions, at 37 °C and under slight agitation (30 r.p.m.). After 80-min incubation, non-adhered bacteria were removed, each well was washed three times with PBS and the remaining adhered bacteria were detached using 0.5% Triton X-100 in PBS. The total amount of initially added and finally adhered bacteria was quantified using flow cytometry. To evaluate the inhibitory effect of a pro- or prebiotic on adhesion of AIEC, the extra 1 ml of PBS was supplemented with 10^8^ probiotic cells per ml or 15 g/l of the prebiotic compound. When co-cultured, the initially added and finally adhered amounts of AIEC were determined using plate counts.

### Short-term AIEC survival and growth inhibition assay

In a second experiment, the inhibitory effect of the selected pro- and prebiotics on the survival/growth of AIEC in nutritional medium (initial pH=6.0) was evaluated during batch incubations. To do this, 15 ml nutritional medium was added to penicillin bottles in which anaerobic conditions were obtained flushing with N2 during 15 cycles of 2 min each at 800 mbar over-pressure and 900 mbar under-pressure. To test the inhibitory effect of the probiotics, 5×10^6^ cells per ml of both the probiotic and AIEC were added. To test the inhibitory effect of prebiotics, 10 g/l prebiotic (control—starch) was added to SHIME nutritional medium containing 5×10^6^ cells per ml of both AIEC and additionally a mixed microbiota from the ascending colon of the SHIME (~10 μl colon suspension). This mixed microbiota was only added during the assays with prebiotics as prebiotic compounds rather inhibit growth of AIEC indirectly through stimulation of other microbes. Penicillin bottles were incubated at 37 °C and 140 r.p.m. for 24 h. At the final time point, pH, D/L-lactic acid, reuterin, 1,3-PDO, SCFA, NH_4_ and counts of AIEC and the probiotic were determined (c.f.u. per ml).

### Dynamic gut model (M-SHIME)

**Figure 3:**
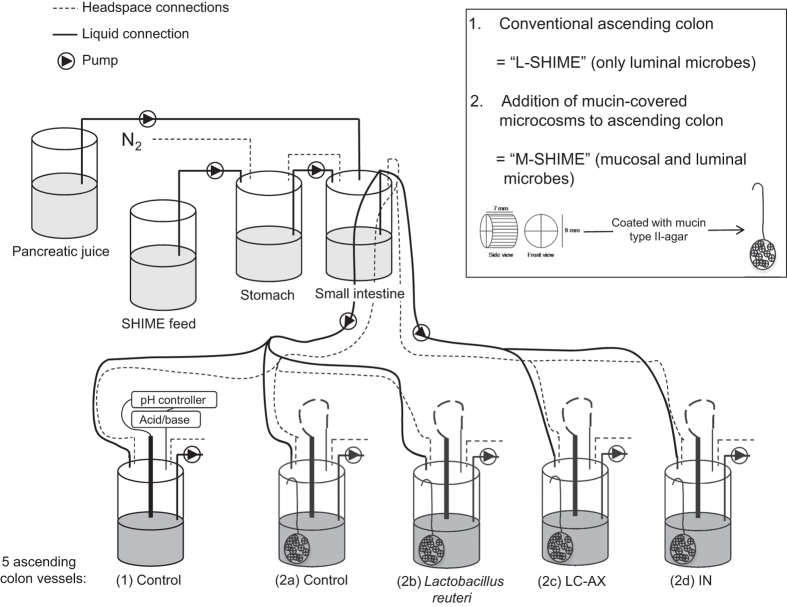
The experimental design was based on the Simulator of the Human Intestinal Microbial Ecosystem (SHIME), a dynamic *in vitro* model of the human gastrointestinal tract. In this experiment, only the first colon compartment (ascending colon) was used and five compartments were run in parallel. The first unit consisted of a conventional setup that only contains luminal microbes (=luminal SHIME or L-SHIME (1)), whereas the other four units were modified by incorporating a mucosal compartment (=mucosal SHIME or M-SHIME (2a) (2b) (2c) (2d)), which contained 20 mucin-covered microcosms per 100 ml suspension. The three last units were treated with *L. reuteri* 1063, LC-AX and IN, respectively.

Recently, we developed an *in vitro* model for the human intestinal tract which accounts for both luminal and mucosal microbes, i.e., the M-SHIME, based on the validated Simulator of the Human Intestinal Microbial Ecosystem (SHIME, Ghent University-ProDigest, Belgium).^19^ The conventional setup comprises five vessels, simulating the stomach, small intestine and three colon regions. In this experiment, only the ascending colon was simulated as this is the most relevant region regarding colonisation of AIEC which is known to predominantly colonise the distal ileum and proximal colon (Figure 3). The mucosal environment in the colon compartments (500 ml) consisted of 80 mucin agar-covered microcosms (AnoxKaldnes K1 carrier, AnoxKaldnes AB, Lund, Sweden). Every 48 h, 50% of the mucin agar-covered microcosms has been replaced by fresh microcosms. The lid of each reactor is slightly opened under constant nitrogen flush and a net containing 40 of the 80 microcosms is quickly replaced by a new one. Old microcosms are then stored for further analysis. The colon regions were simultaneously inoculated with 40 ml of faecal slurry derived from fresh stools of a healthy human volunteer (26 years). Faecal samples were 1:5 (w/v) diluted in phosphate buffer (0.05 M, pH=7) containing 1 g/l sodium thioglycolate, followed by homogenisation with a Stomacher Lab-Blender (Seward Medical, London, UK) and centrifugation to remove particulate material (500 g, 1 min). By imposing identical conditions, similar microbial composition and activity was achieved in the five parallel ascending colon vessels.^36^ Three times per day, 140 ml nutritional medium and 60 ml pancreatic juice were added to the stomach and small intestine, respectively.

### Experimental design M-SHIME study

Based on initial screenings, three treatments were applied in a third experiment, a long-term M-SHIME study with five colon vessels, organised as follows (Figure 3). The first unit consisted of a conventional setup that only simulates luminal microbes (=L-SHIME), while the second also simulates mucosal microbes (=M-SHIME). These two units allowed evaluating the importance of a mucosal environment for the colonisation of AIEC. The M-SHIME unit also served as a control to the other three units that also contained a mucosal environment (=M-SHIME) but that were treated with *L. reuteri* 1063, LC-AX and IN. After inoculation, human faecal microbes were stabilised during 14 days, after which the treatment started. For LC-AX and IN, 3 g per day was added, while for *L. reuteri* 1063 10 ml of an overnight-grown culture, washed in PBS, was applied. On day 22, 23, 24 and 25, 10 ml of an overnight-grown culture of AIEC, washed in PBS, was inoculated. The experiment was stopped at day 31. On the days that the mucosal environment was renewed (day 22, 24, 27, 29 and 31), AIEC was enumerated in the luminal and mucosal microbiota by means of plating.

### Microbial community analysis: plate counts, flow cytometry and DGGE

AIEC numbers were determined by plating on a specific agar, i.e., MacConkey (Oxoid) supplemented with 50 mg/l ampicillin and 20 mg/l erythromycin. Samples were serially diluted in saline solution (8.5 g/l NaCl), after which plates were inoculated and incubated aerobically at 37 °C. Pure cultures of AIEC and probiotic bacteria were quantified using flow cytometry after live–dead staining as described earlier.^19^

DNA extraction according to Boon *et al.*^37^ was performed on the pellet of 1 ml suspension for luminal samples or 0.5 g mucin agar for mucosal samples. Denaturing Gradient Gel Electrophoresis (DGGE) was applied to separate PCR products of the 16S rRNA genes of *Bifidobacterium* sp. and the total bacterial community. For the total community, general bacterial primers 338F-GC and 518R were used,^38^ while the bifidobacteria required a first PCR using specific primers^39^ and a second PCR on the 1:100 diluted PCR product using the general bacterial primers. Polyacrylamide gels (8%) had a denaturing gradient ranging from 45 to 60% for the total microbiota and from 50 to 65% for the bifidobacteria. Gels were run using an Ingeny PhorU apparatus (Ingeny International, Goes, the Netherlands). Normalisation and further analysis was carried out using the BioNumerics software version 5.10 (Applied Maths, Sint-Martens-Latem, Belgium). Specific bands were cut and sequenced by ITT Biotech-Bioservice (Bielefeld, Germany). Sequence data have been submitted to the EMBL database (accession numbers HE985181–HE985183).

### Metabolic activity analysis: lactic acid, SCFA, 3-HPA, NH_4_^+^, 3-HPA and 1,3-PDO

Lactic acid was measured using a D/L-lactic acid kit (R-Biopharm, Mannheim, Germany), according to the manufacturer’s protocols. pH was measured and used to calculate undissociated lactic acid as this is the antimicrobial lactic acid fraction: undissociated lactic acid (%)=1/[1+10^(pH−pKa)]×100%. Further, 3-HPA and 1,3-PDO were quantified by means of HPLC to indicate the reuterin production, and SCFA by means of GC as reported by De Weirdt *et al*.^40^ Using a Kjeltec Auto Distillation (FOSS Benelux, Amersfoort, the Netherlands), NH_4_^+^ in the sample was liberated as NH_3_ by the addition of MgO. Released NH_3_ was distilled from the sample into a boric acid solution, which was back-titrated using a 665 Dosimat (Metrohm, Berchem, Belgium) and 686 Titroprocessor (Metrohm).

### Statistics

All data were analysed using the SPSS 16 software (SPSS, Chicago, IL, USA). Before investigating probability of intergroup differences, normality was studied with a Kolmogorov–Smirnov test. Normal distributed data were further analysed in a one-way ANOVA test, followed by *post hoc* Bonferroni (equal variances) or Dunnett’s T3 (non-equal variances) analysis. Non-normal distributed data were tested for differences using a Kruskal–Wallis with Mann–Whitney test. Differences were considered significant when *P*<0.05.

## Figures and Tables

**Table 1:** The increase in AIEC numbers after 24-h incubation with different *Lactobacillus* sp., as a ratio compared with the initial AIEC count (5×10^6^ c.f.u. per ml) (*n*=3)

	*Control*	*LGG*	*L. reuteri 1063*	*L. mucosae LB2*	*L. acidophilus NCFM*	*P. acidilactici LB1*
AIEC (fold increase)	57.2±19.7^a^	**0.0±0.0**^**b**^	**0.0±0.0**^**b**^	44.3±7.4^a^	**1.3±1.0**^**c**^	**8.8±6.4**^**d**^
						
*Potential markers for AIEC growth*						
Acetate (mM)	4.95±0.67^a^	**0.49±0.86**^**b**^	4.49±0.32^a^	5.01±0.21^a^	**2.99±0.71**^**c**^	**3.24±0.72**^**c**^
Ammonium (mM)	4.52±0.36^a^	**0.08±0.38**^**b**^	**2.85±0.53**^**c**^	6.62±0.21^d^	4.37±0.65^a^	4.64±0.06^a^
						
*Antimicrobial factors*						
D-lactic acid (mM)	0.66±0.17^ab^	0.31±0.04^a^	0.72±0.18^bc^	**1.49±0.14**^**d**^	**1.23±0.31**^**cd**^	**1.78±0.32**^**d**^
L-lactic acid (mM)	0.54±0.14^a^	**4.98±0.17**^**b**^	**1.36±0.12**^**c**^	**1.66±0.17**^**c**^	**2.97±0.44**^**d**^	**3.11±0.35**^**d**^
Acidity (pH)	5.27±0.02^a^	**4.20±0.03**^**b**^	**4.52±0.01**^**c**^	5.34 ±0.01^a^	**4.95±0.21**^**d**^	**4.66±0.10**^**e**^
Undissociated lactic acid (%)	3.7±0.1^a^	**31.5±1.3**^**b**^	**18.0±0.3**^**c**^	3.2±0.1^a^	**7.9±3.4**^**d**^	**13.7±2.8**^**e**^
Undissociated lactic acid (mM)	0.04±0.01^a^	**1.67±0.06**^**b**^	**0.37±0.04**^**c**^	**0.10±0.00**^**d**^	**0.35±0.05**^**c**^	**0.67±0.08**^**e**^
3-HPA (mM)	BDL	BDL	**3.44±0.25**	BDL	BDL	BDL
1,3-PDO (mM)	BDL	BDL	**9.46±0.26**	BDL	BDL	BDL

Abbreviations: AIEC, adherent-invasive *Escherichia coli*; BDL, below detection limit.

Possible antimicrobial factors include D- and L-lactic acid (mM), acidity (pH), corresponding undissociated lactic acid (% and mM) and two markers for reuterin production (3-HPA (mM), 1,3-PDO (mM)). Acetate (mM) and ammonium (mM) are potential markers for AIEC growth. For optimal visualisation, values that indicate antimicrobial effects against AIEC are in bold, while different superscripts indicate significant differences (a, b, c, d or e). BDL, undissociated lactic acid (%)=1/[1+10^(pH−pKa)]×100%.

**Table 2:** The increase in AIEC numbers after 24-h incubation in nutritional medium without (control A) or with addition of an equal amount of a mixed SHIME-derived microbiota (control B) treated with prebiotic compounds (LC-AX, FOS, IN and GOS), expressed as a ratio compared with the initial AIEC count (5×10^6^ c.f.u. per ml) (*n*=5)

	*Control A*	*Control B*	*LC-AX*	*IN*	*FOS*	*GOS*
AIEC (fold increase)	57.2±19.7^a^	**0.27±0.05**^**bc**^	0.44±0.25^bd^	**0.09±0.08**^**c**^	0.60±0.22^d^	**0.10±0.05**^**c**^
						
*Markers for growth of AIEC and/or mixed microbiota*						
Acetate (mM)	4.95±0.67^a^	16.42±0.91^bc^	17.67±1.35^cd^	**15.06±1.55**^**b**^	17.31±0.54^d^	24.87±0.32^e^
Ammonium (mM)	4.52±0.36^a^	7.75±0.44^b^	**6.88±0.40**^**c**^	7.72±0.76^b^	**6.55±0.44**^**c**^	**6.77±0.20**^**c**^
						
*Markers for growth of mixed microbiota*						
Propionate (mM)	BDL	4.74±0.21^a^	**9.98±0.74**^**b**^	4.60±0.35^a^	**7.13±0.15**^**c**^	**6.87±0.27**^**c**^
Butyrate (mM)	BDL	1.62±1.22^a^	0.15±0.21^b^	**1.30±1.60**^**ab**^	BDL	BDL
Total SCFA (mM)	4.95±0.67^a^	22.78±0.89^b^	27.80±1.36^c^	20.95±0.57^d^	24.44±0.66^b^	31.74±0.52^e^
						
*Antimicrobial factors*						
D-lactic acid (mM)	**0.66±0.17**^**ab**^	0.29±0.15^a^	**0.60±0.32**^**ab**^	**0.51±0.44**^**ab**^	**0.81±0.18**^**b**^	**0.70±0.14**^**b**^
L-lactic acid (mM)	0.54±0.14^a^	0.30±0.07^a^	0.42±0.13^a^	0.46±0.25^a^	0.70±0.38^a^	**1.72±0.43**^**b**^
Acidity (pH)	5.27±0.02^a^	4.93±0.02^b^	**4.85±0.01**^**c**^	4.95±0.01^b^	**4.76±0.0**^**d**^	**4.42±0.01**^**d**^
Undissociated lactic acid (%)	3.7±0.1^a^	7.8±0.3^b^	**9.2±0.1**^**c**^	7.5±0.2^b^	**11.2±0.3**^**d**^	**21.5±0.4**^**e**^
Undissociated lactic acid (mM)	0.04±0.01^a^	0.05±0.02^a^	0.09±0.04^a^	0.07±0.05^a^	**0.17±0.03**^**b**^	**0.52±0.08**^**c**^
3-HPA (mM)	BDL	0.56±0.77	1.57±3.51	0.56±1.55	0.28±0.63	1.12±0.62
1,3-PDO (mM)	BDL	**1.83±0.06**^**a**^	1.54±0.05^b^	**1.88±0.75**^**a**^	0.59±0.81^c^	0.61±0.83^bc^

Abbreviations: AIEC, adherent-invasive *Escherichia coli*; BDL, below detection limit; FOS, fructo-oligosaccharides; IN, inulin; LC-AX, long-chain arabinoxylans.

Possible antimicrobial factors include D- and L-lactic acid (mM), acidity (pH), corresponding undissociated lactic acid (% and mM), markers for reuterin production (3-HPA (mM), 1,3-DPO (mM)), markers for metabolic activity of the mixed microbiota (propionate, butyrate and total SCFA (mM)), and two markers for growth of both AIEC and the mixed microbiota (acetate (mM) and ammonium (mM)). For optimal visualisation, values that indicate antimicrobial effects against AIEC are in bold, while different superscripts indicate significant differences between different treatments and/or control A and B (a, b, c, d or e). BDL, undissociated lactic acid (%)=1/[1+10^(pH−pKa)] ×100%.

**Table 3:** The average levels and s.d. of possible antimicrobial factors on time points after AIEC administration during the long-term M-SHIME experiment (day 22–31): D- and L-lactic acid (mM), markers for reuterin production (3-HPA (mM) and 1,3-PDO (mM)) and the amount of bifidobacteria and lactobacilli (log10 c.f.u. per ml), both in lumen and mucus

	*L-Control*	*M-Control*	*M-L. reuteri 1063*	*M-LC-AX*	*M-IN*
*Antimicrobial factors:*					
D-lactic acid (mM)	1.24±0.17^a^	1.61±0.28^ab^	**1.86±0.21**^**b**^	0.81±0.13^c^	0.67±0.15^c^
L-lactic acid (mM)	1.03±0.18^a^	1.23±0.30^ab^	**1.37±0.24**^**b**^	0.72±0.06^c^	0.59±0.10^c^
3-HPA (mM)	BDL	BDL	BDL	BDL	BDL
1,3-PDO (mM)	0.25±0.15^a^	0.44±0.46^a^	**5.63±5.68**^**b**^	**3.38±1.95**^**b**^	**3.11±0.96**^**b**^
					
*Potential antimicrobial factors*					
Lactobacilli (log10 c.f.u. per ml)					
Lumen	3.76±1.27^a^	4.41±0.74^a^	**6.80±0.17**^**b**^	**7.16±0.44**^**b**^	**6.91±0.33**^**b**^
Mucus	—	3.51±1.43^a^	**5.41±0.41**^**b**^	**6.94±0.35**^**b**^	**6.62±0.86**^**b**^
Bifidobacteria (log10 c.f.u. per ml)					
Lumen	6.86±0.24	6.31±0.63	7.05±0.46	6.94±0.76	7.38±0.68
Mucus	—	6.03±0.83^a^	6.43±0.49^b^	**7.59±0.83**^**b**^	**7.56±0.63**^**b**^

Abbreviations: AIEC, adherent-invasive *Escherichia coli*; BDL, below detection limit; IN, inulin; LC-AX, long-chain arabinoxylans.

For optimal visualisation, values that indicate antimicrobial effects against AIEC are in bold, while different superscripts indicate significant differences (a, b or c).
