# Supplementary material for: Arabinoxylans, inulin and Lactobacillus reuteri 1063 repress the adherent-invasive Escherichia coli from mucus in a mucosa-comprising gut model
Source: NPJ Biofilms Microbiomes. 2016 Jul 27;2:16016–. doi: 10.1038/npjbiofilms.2016.16 (PMC5515265; doi:10.1038/npjbiofilms.2016.16)
Supplement: Supplementary Figure 1 [file npjbiofilms201616-s1.ppt]

## Slide 1
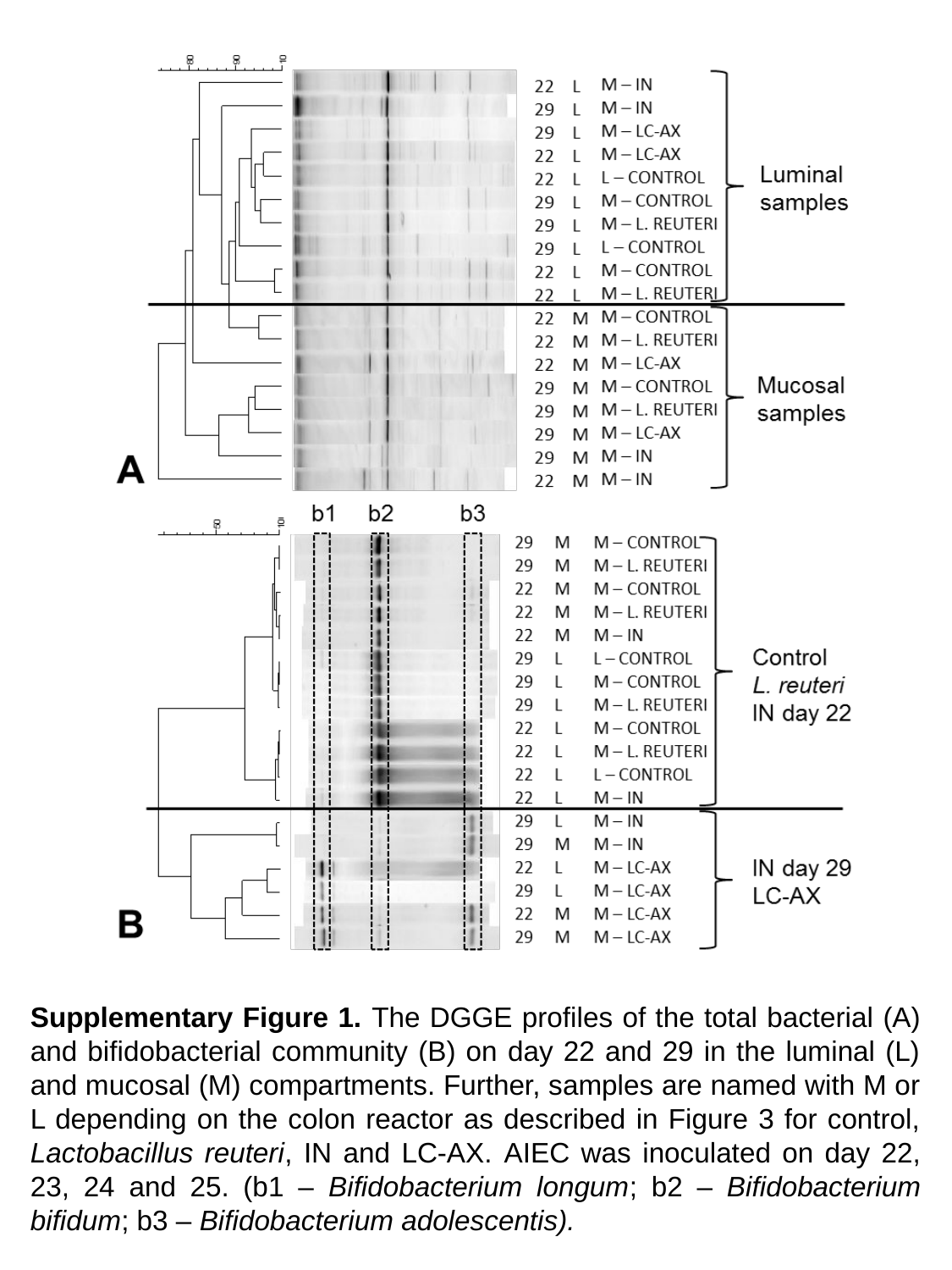

Supplementary Figure 1. The DGGE profiles of the total bacterial (A) and bifidobacterial community (B) on day 22 and 29 in the luminal (L) and mucosal (M) compartments. Further, samples are named with M or L depending on the colon reactor as described in Figure 3 for control, Lactobacillus reuteri, IN and LC-AX. AIEC was inoculated on day 22, 23, 24 and 25. (b1 – Bifidobacterium longum; b2 – Bifidobacterium bifidum; b3 – Bifidobacterium adolescentis).
